# Examining bystander intervention for peer depression and sociodemographic correlates among university students in Singapore

**DOI:** 10.3389/fpsyt.2024.1307807

**Published:** 2024-08-28

**Authors:** Benedict Wei Zhi Lim, Yen Sin Koh, Shazana Shahwan, Chong Min Janrius Goh, Ellaisha Samari, Wei Jie Ong, Kian Woon Kwok, Siow-Ann Chong, Mythily Subramaniam

**Affiliations:** ^1^ Research Division, Institute of Mental Health, Singapore, Singapore; ^2^ School of Social Sciences, Nanyang Technological University, Singapore, Singapore

**Keywords:** anti-stigma intervention, ARTEMIS, attitudes, bystander intervention, depression, Singapore, university students

## Abstract

**Introduction:**

The Advancing Research To Eliminate Mental Illness Stigma (ARTEMIS) study evaluated the impact of an intervention developed and implemented in Singapore on attitudes towards depression in university students. We aimed to assess the likelihood of university students intervening when their peers suffer from depression, before and after the ARTEMIS intervention.

**Methods:**

390 students were recruited from a university in Singapore. The ARTEMIS intervention comprised a lecture by a trained mental health professional, a sharing session by a person with lived experience of depression, and a question-and-answer segment with a panel. The Bystander Intervention Scale for Depression (BISD) was administered at baseline, post-intervention, and 3-month follow-up. BISD assessed four factors: *acceptance of responsibility to intervene*, *knowledge on how to intervene*, *awareness of depression among peers*, and *vigilance towards possible symptoms of depression*. Linear mixed models were conducted to investigate associations. Sociodemographic correlates were also examined.

**Results:**

A favourable shift in all factors was observed at post-intervention, which weakened at 3-month follow-up. Having past experience in the mental health field (β=1.50) and older age (β=0.18) were significantly associated with *knowledge on how to intervene*. Having social contact with mental illness (SCMI) and past experience in the mental health field (PEMHF) were significantly associated with *awareness of depression among peers* (SCMI β=0.89, PEMHF β=0.43) and *vigilance towards possible symptoms of depression* (SCMI β=0.39, PEMHF β=0.61).

**Discussion:**

The short-term results of the intervention appeared promising across all BISD factors; however, these results were not sustained after 3 months. Future research should include the impact of ‘booster’ interventions over time. Sociodemographic factors that were identified to be significant correlates should also be considered when planning for future interventions.

## Introduction

Depression is a common but severe mental illness, with the World Health Organisation (WHO) global report estimating that nearly 4.4% of the world’s population was living with depressive disorders (1). The Global Burden of Disease Study 2019 showed that approximately 970.1 million individuals worldwide suffer from mental illnesses, of which 279.6 million suffer from depressive disorders (2). To compound the high global prevalence of mental illness, stigma afflicts people with mental illness worldwide (3). Crocker et al. (4) proposed that stigmatisation occurs when a person possesses (or is believed to possess) “some attribute or characteristic that conveys a social identity that is devalued in a particular social context” (p. 505) (5). People with mental illness face stigmatisation in many aspects of their lives, including their social lives, careers and in academic settings. Stigma has been shown to deter people with mental illness from seeking treatment and from advocating for better mental health services, resulting in a global treatment gap (6, 7).

Singapore is also beset by similar issues of a high prevalence of depression and stigmatisation of people with mental illness. The Singapore Mental Health Study 2016 (SMHS 2016) established the prevalence of mental disorders as 13.9% (n=6126), with Major Depressive Disorder (MDD) being the most common mental illness in Singapore, with a prevalence rate of 6.3% (8). Local research has also indicated significant and widespread stigma against people with mental illness (9–11).

A study by Vaingankar et al. (12) suggested that young adults in Singapore are more likely to develop mental illness than those belonging to other age groups. They identified the age of onset of any mental illness in Singapore to be 22 years, and 26 years for depression (12). The SMHS 2016 also identified the lifetime prevalence of mental illness in individuals aged 18–34 to be the highest at 21.6% and that a large treatment gap existed in this group (8). Subramaniam et al. (13) further identified poor mental health literacy and stigmatising attitudes toward mental illness as key factors that contributed to the treatment gap. They also identified label avoidance and fear of jeopardizing job prospects as reasons for treatment avoidance among Singaporeans with higher education, which may also help explain treatment avoidance among the youth, as most of whom would have undergone higher education, given Singapore’s emphasis on meritocracy (13). As such, research evidence suggests that reducing mental health stigma in youth might bridge the treatment gap, facilitating early intervention and reducing the potential for greater negative impact due to cases going unnoticed and untreated.

Schomerus et al. (14) found that while public understanding of mental health has increased over the years, the level of stigma toward mental illness remains constant. This finding suggests that mental health literacy alone is insufficient for reducing mental health stigma. Anti-stigma initiatives have been implemented worldwide through various strategies (education, contact, and protest) targeted at different populations (students, military, healthcare professionals, etc.) (15–17). While many anti-stigma initiatives have also been implemented in Singapore, there is a significant lack of research evaluating these initiatives (18). The Advancing Research To Eliminate Mental Illness Stigma (ARTEMIS) was conducted to evaluate the effectiveness of an anti-stigma intervention that focused on depression among university students in Singapore (19, 20). The ARTEMIS study aimed to implement and evaluate an intervention that was designed to improve: (1) recognition of depression, (2) attitudes towards individuals with mental illness, (3) willingness to interact with people with mental illness, and (4) bystander attitudes. The term “bystander attitudes” (otherwise referred to as “bystander apathy” or “bystander effect”) refers to the theory that an individual’s likelihood of rendering assistance to someone in need decreases as the number of bystanders increase (21). To put it simply, if someone thinks other people are there to help or will help, the individual himself or herself is then less likely to help.

Darley and Latane (22) posited a five-step model of intervention in an emergency: (1) noticing that something is wrong; (2) defining the event as an emergency; (3) deciding on the degree of personal responsibility; (4) determining the specific mode of intervention; and (5) implementing the intervention. Peer involvement, in this case, can thus be likened to bystander intervention, where a person’s involvement in another’s mental health situation is dependent on whether they deem it to be concerning and feel responsible to intervene. Examining peer involvement would allow for greater insights into the behavioural tendencies of youth when dealing with a peer with mental illness, and this could help inform future anti-stigma research or efforts.

Therefore, the current study aimed to (1) assess the likelihood of university students intervening when their peers suffer from depression at post-intervention and three months later and (2) examine the various sociodemographic correlates of those who were most likely to intervene as a result of the intervention that was carried out in the ARTEMIS study.

## Methodology

### Sampling and recruitment

Data was collected as part of the ARTEMIS study, which was a longitudinal study conducted in collaboration with researchers from the Institute of Mental Health (IMH) and a local university in Singapore. The researchers reached out to students and recruited them via (1) invitation emails from the school with a weblink to register their participation; (2) a post on the university’s Facebook page with a similar weblink; and (3) posters put up with QR codes that lead to the same website for registration. Participants were required to be (1) 18 to 35 years of age; (2) a student of the university at the point of registration; and (3) literate in English (the only language used in the intervention). After registering their interest, students were sent a consent form via email, and parental consent was also obtained for students under the age of 21 years (age of minority in Singapore). On the day of the session, research staff also obtained written informed consent from participants and clarified any doubts they had. A total of 392 participants were recruited this way. However, the final analysis only used the data from 390 participants, with 2 participants being excluded (due to age being above the eligibility criteria and incomplete survey data, respectively). The study received ethics approval from the National Healthcare Group Domain Specific Review Board (NHG-DSRB, reference number: 2018/00695).

### Intervention

The ARTEMIS intervention utilized the concepts of *education* and *contact* based on the stigma-reduction theory by Corrigan and Penn (23). They posited that the core aspects of the stigma-reduction theory are (1) *protest* – identify and speak out against inaccurate and negative portrayals and beliefs about mental illness; (2) *education* – provide more information about mental illness so that the public can have a more informed opinion about it, as well as people with mental illness; and (3) *contact* – interaction with people with mental illness to challenge any existing stereotypes regarding these people (23). ARTEMIS was a single-arm intervention with pre-post intervention evaluation and an additional 3-month follow-up. The study lasted from October 2018 to April 2019. The intervention was delivered in a single session, with a total of nine sessions conducted to accommodate all participants. The sessions, each lasting around 50–60 minutes, were conducted in the evening (to avoid scheduling conflicts) with a maximum of 50–80 participants at a time (depending on venue size and to keep sessions interactive).

The intervention comprised: (1) a 30-minute lecture on depression by a trained mental health professional, which was supplemented by a PowerPoint presentation and a WHO video titled “I had a black dog, his name was depression” created by Matthew Johnstone depicting his personal struggles with depression (https://www.youtube.com/watch?v=XiCrniLQGYc). The lecture educated participants on the prevalence, symptoms and biopsychosocial causes of depression, as well as treatment options and help-seeking avenues. The intervention included a contact component; (2) a 10-minute sharing session by someone with lived experience of depression where she detailed the clinical aspects of her mental illness, her challenges regarding accepting her illness and help-seeking, as well as her road to recovery. Lastly, the intervention constituted (3) a 10-minute Question and Answer (Q&A) segment with a panel comprising a senior consultant psychiatrist, a mental health research expert, and the person with lived experience, which allowed students to clarify any doubts regarding the presentation, sharing session or any general queries regarding mental health. A more detailed description of the intervention can be found in Subramaniam et al. (20). Data was collected through a series of questionnaires at three time points: Pre-intervention, post-intervention and 3 months after the intervention had concluded. Pen and paper questionnaires were used for pre- and post-intervention, while emails with links to an online questionnaire (via QuestionPro) were sent to participants at the 3-month follow-up.

### Measures

#### Bystander intervention scale for depression

The BISD was used to assess the likelihood of participants intervening when their peers showed signs of depression. The scale was developed in-house by the IMH research team based on the bystander intervention model by Latane and Darley (22, 24). It is a 17-item self-reported questionnaire that measures the respondent’s bystander attitudes across four factors: (1) Awareness of depression among peers, (2) Vigilance towards possible symptoms of depression, (3) Knowledge on how to intervene and (4) Acceptance of responsibility to intervene. The response options for each item comprised a 5-point Likert scale ranging from “1-Strongly agree” to “5-Strongly disagree”. Items under *Vigilance towards possible symptoms of depression* were reverse coded, and scoring for each domain was done by summing all item scores for that domain. To allow for more intuitive interpretations of the summed scores, all items were reverse scored such that the ranges became “1-Strongly disagree” to “5-Strongly agree” (items under factor 2 remain reverse coded from the rest). Therefore, higher scores in (1), (3), and (4) suggested that participants had more favourable bystander attitudes and could be seen as more likely to intervene when their peer had depression, while the reverse was true for (2). The internal consistencies of factors 1 and 2 were considered satisfactory, while those of factors 3 and 4 were considered low (25).

(Refer to the **Appendix** for the table of Cronbach’s alpha values for the four factors.)

Ong et al. (24) conducted multiple rounds of exploratory factor analysis (EFA) on the BISD to examine its underlying factor structure, which resulted in the removal of 3 items (one for low factor loading and two for cross-loading) (23). As such, this study only analysed the 14 remaining items. The ranges of scores (min-max) for the 14 items across the four factors are as follows (1) 3–15, (2) 2–10, (3) 4–20, and (4) 5–25.

(Refer to the **Appendix** for the list of BISD items.)

#### Sociodemographic information

Participants were required to provide sociodemographic information, including gender, ethnicity, age, year of study, and course of study. Participants were also asked about their exposure to mental illness using two questions that inquired whether they had any social contact (close friends or family members) with mental illness and their previous experience within the mental health field.

### Statistical analysis

Statistical analysis was conducted using IBM SPSS Statistics 23 and Stata M/P Version 17, using a two-sided test at a 5% significance level. Means and standard deviations were presented for the scores for bystander effects across time. Frequencies and percentages were reported for categorical variables. Linear mixed models (LMM) were used to examine the impact of the ARTEMIS intervention on bystander attitudes among university students. The LMM was suitable because it accounts for missing data, individual heterogeneity, and any possible intra-individual correlation in repeated measurements over time. A time variable was also included as a fixed and random effect in the LMM to account for intra-individual and inter-individual variation in the outcomes over time. The models were first generated without including any covariates to compare the BISD scores across three time points (pre-intervention, post-intervention, and at 3-month follow-up). Linear and quadratic effects across time were tested for and included in the model because they were significant (tested using the likelihood ratio test). Sociodemographic covariates (Gender, ethnicity, age, course of study, whether close friends or family members have a mental illness, and past experience within the mental health field) were included in the models. Age was mean-centred to reduce multicollinearity, which in turn allowed for easier interpretation of the coefficients (26). Interaction terms between time and each covariate were explored to examine the effect of potential covariates on the change in bystander effect over time. The model includes only significant interaction terms, which were assessed using the likelihood ratio test, Akaike information criterion (AIC), and Bayesian information criterion (BIC). Finally, effect sizes were also calculated using Cohen’s D, with the following formula:


$$Cohen^{’}s\;D=\;\frac{Difference\;between\;the\;mean\;of\;the\;two\;timepoints}{Standard\;deviation\;of\;the\;difference}$$

The effect sizes can be interpreted using: 0.2–0.5 (small); 0.5–0.8 (medium); and 0.8 or higher (large) (27).

## Results

### Sample characteristics

The sample characteristics are presented in **Table 1**. All missing data was dealt with by removal in a listwise manner. The baseline sample and post-intervention sample consisted of 390 participants ranging from 18–31 years of age (mean age = 22.28 years). The majority were female (60.3%) and Chinese (82.8%). 42.6% of the sample had close friends or family with mental illness and 22.2% had past experience in the mental health field. A total of 324 participants completed the 3-month follow-up survey (retention rate = 83.1%). Similarly, the majority were female (60.8%) and Chinese (84.0%). Of the 324 participants, 41.4% knew someone close with mental illness and 23.3% had past experience in the mental health field.

**Table 1:** Sociodemographic characteristic at different time points.

Variable	Pre-intervention and Post-intervention (n = 390) n (%)	3-month (n = 324) n (%)
Mean age (SD)	22.28 (2.26)	22.25 (2.24)
Gender		
Female	235 (60.3%)	197 (60.8%)
Male	155 (39.7%)	127 (39.2%)
Ethnicity		
Chinese	323 (82.8%)	272 (84.0%)
Malay	12 (3.1%)	9 (2.8%)
Indian	37 (9.5%)	32 (9.9%)
Others	18 (4.6%)	11 (3.4%)
Having close friends or family members with a mental illness		
No	224 (57.4%)	190 (58.6%)
Yes	166 (42.6%)	134 (41.4%)
Past experience within the mental health field		
No	301 (77.8%)	247 (76.7%)
Yes	86 (22.2%)	75 (23.3%)
Course of study		
Non-STEM	160 (41.2%)	132 (40.9%)
STEM	228 (58.8%)	191 (59.1%)
Year of study		
Year 1	130 (33.3%)	103 (31.8%)
Year 2	97 (24.9%)	86 (26.5%)
Year 3	79 (20.3%)	67 (20.7%)
Year 4	84 (21.5%)	68 (21.0%)

Missing Data: Past experience within the mental health field (n for pre- and post-intervention = 3, n for 3-month = 2), Course of study (n for pre- and post-intervention = 2, n for 3-month = 1).

STEM, science, technology, engineering, and math.

### BISD scoring

**Figure 1:**
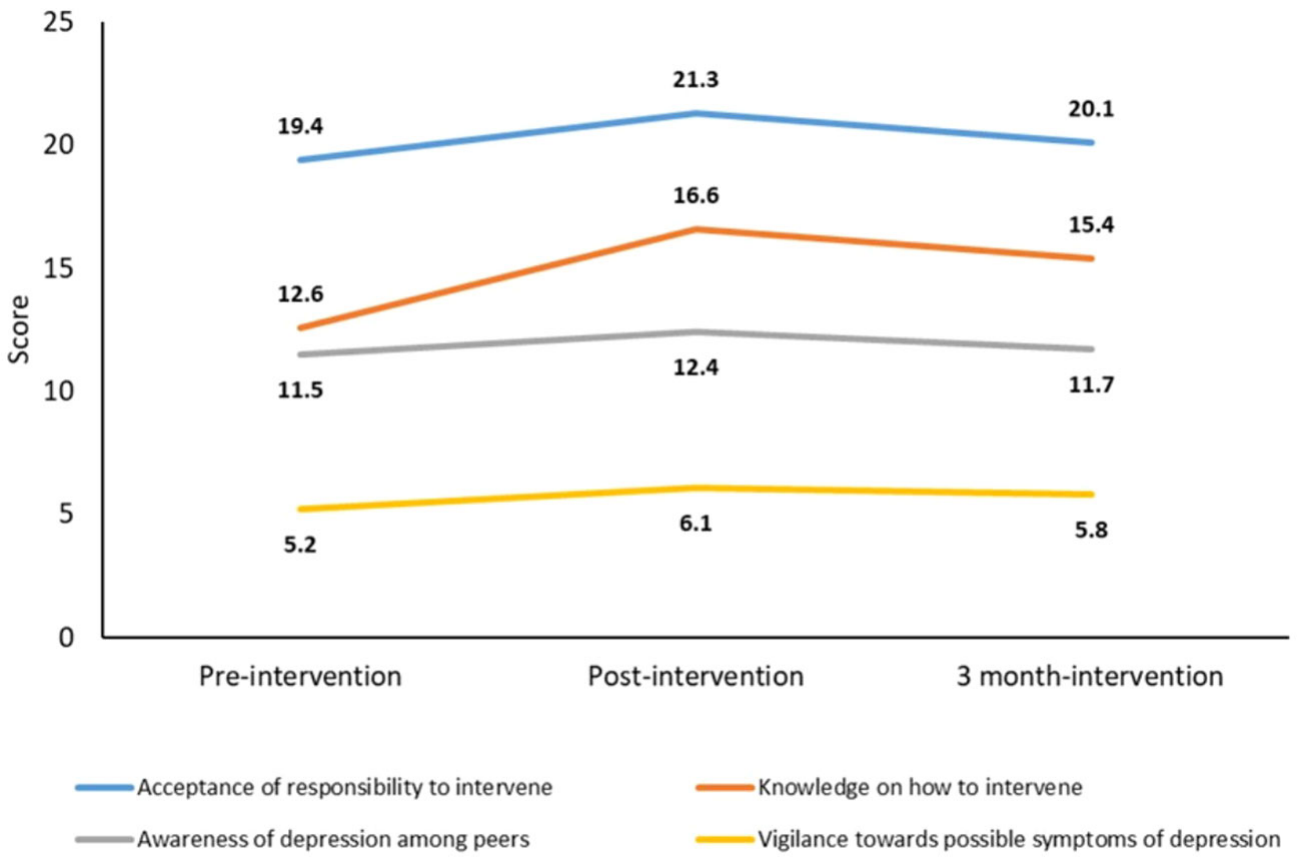
Mean factor scores across all three intervention time-points.

Linear and quadratic effects were significant across all four factors, indicating that scores in all factors increased at post-intervention and slightly decreased after 3 months, as illustrated in **Figure 1**. **Table 2** highlights the significant differences in scores between time points that were observed in all factors when comparing (1) pre-intervention to post-intervention, (2) post-intervention to 3-month follow-up, and (3) pre-intervention to 3-month follow-up. However, there was no significant difference in scores when comparing pre-intervention to 3-month follow-up scores for *Awareness of depression among peers* (p=0.074).

**Table 2:** Scores for bystander effect at different time points.

	Pre-intervention	Post-intervention	Effect Size ^1^	p-value ^1^	3-month intervention	Effect Size ^2^	p-value ^2^	Effect Size ^3^	p-value ^3^
	Mean (SD)	Mean (SD)	Cohen’s D		Mean (SD)	Cohen’s D		Cohen’s D	
Factor 1: Awareness of depression among peers	11.53 (1.99)	12.37 (1.94)	0.51	< 0.001	11.71 (1.94)	0.37	< 0.001	0.11	0.074
Factor 2: Vigilance towards possible symptoms of depression	5.20 (1.50)	6.11 (1.81)	0.49	< 0.001	5.81 (1.66)	0.17	0.001	0.33	< 0.001
Factors 3: Knowledge on how to intervene	12.56 (3.32)	16.57 (2.14)	1.27	< 0.001	15.38 (2.63)	0.52	< 0.001	0.85	< 0.001
Factor 4: Acceptance of responsibility to intervene	19.39 (2.81)	21.33 (2.57)	0.75	< 0.001	20.06 (2.62)	0.58	< 0.001	0.24	< 0.001

^1^ Pre- vs post-intervention, ^2^ post vs 3-month intervention, ^3^ pre- vs 3-month intervention. All p-values were obtained from unconditional linear mixed model.

Missing data: Factor 1 at pre intervention (n = 2), Factor 1 at post-intervention (n = 2), Factor 2 at pre-intervention (n = 1), Factor 3 at pre-intervention (n = 2), Factor 3 at post-intervention (n = 1), Factor 4 at pre-intervention (n = 2).

### Awareness of depression among peers

The effect size was medium at post-intervention (0.51) and small at 3-month follow-up (0.37) (**Table 2**). Age (β=-0.09, 95% CI: −0.18 to −0.004), having social contact with mental illness (β=0.89, 95% CI: 0.56 to 1.22), past experience in the mental health field (β=0.43, 95% CI: 0.04 to 0.82) and year of study (β=0.27, 95% CI: 0.11 to 0.43) were all significantly associated with *Awareness of depression among peers* (**Table 3**).

**Table 3:** Regression results on the correlates associated with the bystander effect.

	Factor 1: Awareness of depression among peers		Factor 2: Vigilance towards possible symptoms of depression		Factor 3: Knowledge on how to intervene		Factor 4: Acceptance of responsibility to intervene	
	β (95% CI)	p-value	β (95% CI)	p-value	β (95% CI)	p-value	β (95% CI)	p-value
Time	1.55 (1.25 to 1.85)	**< 0.001**	1.77 (1.40 to 2.14)	**< 0.001**	8.38 (7.54 to 9.23)	**< 0.001**	3.50 (3.09 to 3.91)	**< 0.001**
Time^2^	−0.73 (−0.88 to −0.59)	**< 0.001**	−0.72 (−0.90 to −0.54)	**< 0.001**	−3.47 (−3.88 to −3.07)	**< 0.001**	−1.59 (−1.79 to −1.39)	**< 0.001**
Age (years)	−0.09 (−0.18 to −0.004)	**0.041**	0.09 (0.02 to 0.16)	**0.012**	0.18 (0.07 to 0.29)	**0.002**	−0.08 (−0.22 to 0.05)	0.236
Female vs male	0.21 (−0.16 to 0.58)	0.268	−0.18 (−0.48 to 0.11)	0.224	0.60 (−0.05 to 1.24)	0.070	0.07 (−0.43 to 0.57)	0.789
Malay vs Chinese	0.56 (−0.35 to 1.47)	0.229	−0.37 (−1.11 to 0.36)	0.318	0.71 (−0.48 to 1.90)	0.244	0.63 (−0.60 to 1.87)	0.316
Indian vs Chinese	0.27 (−0.28 to 0.81)	0.340	−0.02 (−0.46 to 0.42)	0.925	0.52 (−0.19 to 1.23)	0.151	0.86 (0.13 to 1.60)	**0.022**
Others vs Chinese	0.05 (−0.73 to 0.83)	0.903	−0.60 (−1.22 to 0.03)	0.060	−0.40 (−1.43 to 0.63)	0.443	0.27 (−0.79 to 1.32)	0.620
Having close friends or family members with a mental illness (Yes vs No)	0.89 (0.56 to 1.22)	**< 0.001**	0.39 (0.13 to 0.66)	**0.004**	−0.11 (−0.54 to 0.32)	0.604	0.66 (0.22 to 1.11)	**0.004**
Past experience within the mental health field (Yes vs No)	0.43 (0.04 to 0.82)	**0.029**	0.61 (0.23 to 0.99)	**0.002**	1.50 (0.79 to 2.21)	**< 0.001**	1.04 (0.52 to 1.57)	**< 0.001**
Year of Study	0.27 (0.11 to 0.43)	**0.001**	0.04 (−0.09 to 0.17)	0.536	0.10 (−0.11 to 0.30)	0.367	−0.05 (−0.26 to 0.17)	0.667
Course of Study (STEM vs Non-STEM)	−0.32 (−0.66 to 0.02)	0.064	0.04 (−0.23 to 0.32)	0.754	0.03 (−0.59 to 0.65)	0.915	−0.27 (−0.74 to 0.19)	0.243
Interaction Terms								
Time x Age (years)							0.29 (0.11 to 0.47)	**0.002**
Time^2^ x Age (years)							−0.09 (−0.17 to 0.0007)	0.052
Time x Male					−1.18 (−2.16 to −0.21)	**0.017**		
Time^2^ x Male					0.59 (0.12 to 1.05)	**0.013**		
Time x Past experience within the mental health field			−1.14 (−1.91 to −0.36)	**0.004**	−2.59 (−3.72 to −1.47)	**< 0.001**		
Time^2^ x Past experience within the mental health field			0.49 (0.11 to 0.87)	**0.011**	1.07 (0.54 to 1.61)	**< 0.001**		
Time x STEM					−1.37 (−2.34 to −0.40)	**0.006**		
Time^2^ x STEM					0.72 (0.26 to 1.19)	**0.002**		

Values in bold indicate statistically significant p-values.

### Vigilance towards possible symptoms of depression

The effect size was small at both post-intervention (0.49) and 3-month follow-up (0.17) (**Table 2**). Age (β=0.09, 95% CI: 0.02 to 0.16), having social contact with mental illness (β=0.39, 95% CI: 0.13 to 0.66), and past experience in the mental health field (β=0.61, 95% CI: 0.23 to 0.99) were significantly associated with *Vigilance towards possible symptoms of depression*. Linear effects showed that students with past experience in the mental health field had a smaller increase in their scores for this factor at post-intervention (β=−1.14, 95% CI: −1.91 to −0.36). Quadratic effects showed that students with past experience in the mental health field also had a smaller decrease in scores at 3-month follow-up (β=0.49, 95% CI: 0.11 to 0.87) (**Table 3**).

### Knowledge on how to intervene

The effect size was large at post-intervention (1.27) and medium at 3-month follow-up (0.52) (**Table 2**). Among the sociodemographic covariates, age (β=0.18, 95% CI: 0.07 to 0.29) and having past experience in the mental health field (β=1.50, 95% CI: 0.79 to 2.21) were significantly associated with *Knowledge on how to intervene*. Linear effects showed that male students had a smaller increase in their scores at post-intervention (β=−1.18, 95% CI: −2.16 to −0.21), as did students with past experience in the mental health field (β=−2.59, 95% CI: −3.72 to −1.47) and students in the Science, Technology, Engineering and Math (STEM) courses (β=−1.37, 95% CI: −2.34 to −0.40). Quadratic effects showed that male students (β=0.59, 95% CI: 0.12 to 1.05) and students in the STEM courses (β=0.72, 95% CI: 0.26 to 1.19) had a smaller decrease in their scores at 3-month follow-up, while students with past experience in the mental health field showed a larger decrease in scores at 3-month follow-up (β=1.07, 95% CI: 0.54 to 1.61) (**Table 3**).

### Acceptance of responsibility to intervene

The effect size for this factor was medium at both post-intervention (0.75) and 3-month follow-up (0.58) (**Table 2**). **Table 3** indicates that ethnicity (β_Indian vs Chinese_=0.86, 95% CI: 0.13 to 1.60), having social contact with mental illness (β=0.66, 95% CI: 0.22 to 1.11), and having a past experience in the mental health field (β=1.04, 95% CI: 0.52 to 1.57) were significantly associated with *Acceptance of responsibility to intervene*. Linear effects also showed that older students had a smaller increase in their scores from pre-intervention to post-intervention (β=0.29, 95% CI: 0.11 to 0.47). No significant interaction with age was found at a 3-month follow-up.

## Discussion

Our study examined the impact of the ARTEMIS intervention on bystander attitudes toward peer depression. We observed significant shifts in bystander attitudes across two time points (post- and 3-month follow-up). A favourable change in attitudes was observed between pre-intervention and post-intervention for all BISD factors, as evidenced by the increase in scores. However, this favourable change in scores was not sustained, as shown by the decrease in scores from post-intervention to 3-month follow-up (**Figure 1**). Subramaniam et al. (20) observed a similar trend in terms of participants’ depression literacy and personal stigma.

We posit that reducing stigma against people with mental illness through ARTEMIS increased the willingness of participants to help their peers with depression, which suggested an improvement in bystander attitudes. The favourable short-term results suggested that the ARTEMIS intervention, through education and contact, was effective at changing bystander attitudes for the better. A lack of mental health literacy has been identified as a contributor to stigmatising attitudes against people with mental illness (28, 29). Educational intervention has, in turn, been shown to reduce mental health stigma (16, 30). The educational component of ARTEMIS emphasized a biopsychosocial model for depression, thereby influencing the way participants view depression, by bringing in an element of biological attribution. Han et al. (31) identified “biological attribution” as a factor for help-seeking behaviour and suggested that biological education to legitimize depression as a disease entity would increase people’s motivation to seek help. Mechanic et al. (32) found that people with mental illness who attributed their problems to a “physical, medical or biological” problem instead of a “mental illness” reported better social relations and a higher quality of life. The intervention might have led participants to view depression as containing a biological component, which may have caused them to view depression through a less stigmatising frame, thereby increasing the likelihood of helping a peer with depression.

Increasing mental health literacy may not be sufficient in combating stigma, which means that education must be complemented with other components, such as contact (15). The contact component of ARTEMIS intervention aimed to foster a positive relationship between those with depression and those without by fulfilling the “equal status” condition of Allport’s Intergroup Contact Theory, which posited that contact between groups under optimal conditions could effectively reduce intergroup prejudice (33). Research has since demonstrated the effectiveness of contact-based interventions at reducing stigma, sometimes even more so than education, although this has been disputed (34–36). By involving participants in a sharing session by a person with lived experience of depression, ARTEMIS aimed to reduce their depression stigma and equalize the status between the two groups. Given the improvement of BISD scores between baseline and post-intervention, ARTEMIS has, through education and contact, demonstrated its effectiveness at improving university students’ bystander attitudes, thereby increasing their likelihood of intervening when their peers suffer from depression.

However, another significant shift between post-intervention to the 3-month follow-up tells a less-than-favourable story, with a decline in scores for all factors at the 3-month follow-up. A comparison between the baseline and the 3-month follow-up suggested a decrease in BISD scores toward initial levels. Thus, the effects of ARTEMIS on bystander attitudes, while promising at first, were not sustained after 3 months, although the 3-month follow-up scores were still higher than baseline, suggesting that ARTEMIS was most effective in the short term, but the benefits of the intervention appear to be unsustainable in the long-term. Other anti-stigma initiatives have indicated similar trends and literature on the longer-term impact of such initiatives remains limited (17, 18, 37). Considering such trends, we posit that “attitude strengths” could explain the decrease in BISD scores at 3-month follow-up. Krosnick and Petty (38) defined the term as an attitude’s durability and impact, where durability refers to (1) the persistence of attitude and (2) its resistance to attack, while impact refers to (3) how said attitude influences information processing and judgement, as well as (4) how it can guide behaviours. In the context of bystander attitudes, stigmatizing attitudes toward people with mental illness have high durability and impact, meaning that such attitudes are not only deep-rooted and highly resistant to change (durable), but they also prevent individuals from helping others with mental illness (impactful). Research has also established a correlation between attitude strength and the likelihood of persuasion (39). As ARTEMIS was a single-session intervention, participants’ longstanding attitudes towards bystander intervention for peer depression may have outlasted the temporary effects of persuasion, which could have counteracted the positive impacts of ARTEMIS and caused BISD scores to decrease after 3 months.

Several sociodemographic factors were significantly associated with BISD factors, including age, ethnicity, having social contact with mental illness and having past experience in the mental health field. Our analysis suggested that older students were more vigilant to the symptoms of depression and had more knowledge of how to intervene, but they were also less aware of depression among peers. Our observation regarding older students having greater vigilance and higher knowledge is consistent with existing literature (40, 41). We posit that older students (or “mature students”), in this case either senior undergraduate or graduate students, were more likely to have developed proper coping strategies that included building their knowledge on depression recognition and professional help seeking avenues. Given the age range of participating students (18–31) versus that of the average university student in Singapore (18–24), we also posit that the older students, compared to their junior or undergraduate counterparts, have moved on to a different stage of their studies, which involve less participation in group activities in and beyond the formal curriculum. They might also have lesser peers due to an age disparity and generational gap which resulted in them being socially isolated from their younger peers. Research has shown that mature students have a higher tendency to be socially isolated from peers, as compared to their more traditionally-aged counterparts (42, 43). This could potentially explain their lower awareness of depression symptoms in peers, although it warrants further investigation, as older students might also have other commitments (like work or family) which limit their time spent on campus. Graduate students are also known to focus heavily on components of their programmes such as laboratory work, library research, or fieldwork, as well as in the writing of academic papers and dissertations.

Indian participants were also more likely to accept the responsibility to intervene, as compared to Chinese participants, and this finding is consistent with findings by Yuan et al. (9) and Subramaniam et al. (11), where both studies identified Indian participants as being more tolerant and accommodating toward people with mental illness. The Chinese cultural concept of “face”, a marker of social standing, may help explain why Chinese participants were more hesitant to accept the responsibility to intervene (44). Given the stigma surrounding mental illness, Chinese participants might be afraid of losing “face”. The fear of losing “face” is akin to the fear of stigma by association, where Chinese participants anticipate shame for associating with or helping someone with mental illness and were, therefore, less likely to assume the responsibility of helping their depressed peers. Our observation of Indian participants did not extend to the Malay and Other ethnicity participant groups. By using samples that are more representative of the population, future research could potentially identify similar significant trends in these groups. Using qualitative measures could also help identify possible links between ethnicity and bystander intervention.

Participants who had social contact with mental illness were found to be more vigilant about possible symptoms of depression and were more aware of any peers who might be depressed. They were also more likely to accept the responsibility to intervene when encountering a depressed peer. Previous studies have also identified close social contact as a predictor of less stigmatising attitudes toward people with mental illness (45, 46). It is possible that those who had social contact would better understand the realities of dealing with depression and would therefore be less likely to stigmatise and more likely to intervene should their peers suffer the same problem. Participants with past experience in the mental health field were similar, except that they were also more knowledgeable on how to intervene. They might have undergone more training or exposure that would have equipped them with the appropriate competencies and skillsets, thereby giving them the self-efficacy or confidence to intervene.

### Implications

To strengthen any short-term positive changes in attitudes, future interventions can consider ‘booster’ interventions (17). The inoculation theory, proposed by Mcguire (47, 48), suggests that beliefs and attitudes can be strengthened against challenges in the same way that an immune system is strengthened against diseases by exposing them to weak attacks and allowing them to take an active role in building their defences. A meta-analysis of 54 studies utilizing inoculation theory was conducted by Banas and Rains (49), which supported its effectiveness. Therefore, future interventions can consider using methods such as introducing debates to allow participants to take an active role in strengthening their newfound post-intervention attitudes, compared to more conventional methods like lectures. Omelicheva and Avdeyeva (50) conducted an experiment to compare the effectiveness of lectures and debates by presenting controversial topics in both forms. Participants were made to fill out pre- and post-test surveys that captured their interests, concerns, and attitudes toward the topics. The researchers found that debates were more effective than lectures in developing students’ comprehension of complex concepts and application and critical evaluation skills, although lectures were more effective at disseminating factual knowledge. Thus, introducing a debate component into ARTEMIS could supplement the lecture and potentially bring about greater change in bystander attitudes.

We also identified several sociodemographic factors that significantly correlate to the BISD factors, which could inform healthcare policymaking for specific population subgroups and future planning for anti-stigma initiatives. For example, universities can develop more initiatives that enable their older students to better integrate with the rest of the student body, which provides more exposure to peers suffering from depression. This greater integration would allow the older students to better look out for depressive signs among their younger counterparts, and for the younger students to learn from their older peers, thereby benefitting the student body as a whole. Additionally, public resources can be invested in educational initiatives to reduce certain ingrained barriers to intervention, such as a fear of doing more harm than good or a fear of negative social consequences for helping. A study by Bennett, Banyard, and Garnhart (51) on bystander intervention in the context of sexual violence found that 31.5% of respondents identified *Failure to take intervention responsibility* (of which *Fear of further harm to the victim* is a subdomain) and 13.6% identified *Failure to intervene due to audience inhibition* as barriers to intervention. Addressing these barriers in the context of mental illness could improve bystander attitudes and reduce the effects of certain cultural barriers, such as the Chinese concept of “face”. Finally, given the significance of social contact with mental illness on bystander attitudes, future anti-stigma initiatives could also be designed with this in mind, perhaps by utilizing contact sessions with persons with mental illness to reduce the stigma surrounding them.

### Strengths and limitations

The ARTEMIS intervention is built on a reliable theory of stigma reduction by Corrigan and Penn (23). By employing the biopsychosocial model, the intervention also addresses stigma reduction across all main facets of human behaviour (biological, psychological and social). This is something that other methodologies or interventions may not have considered. Compared to other interventions, ARTEMIS combines both education and contact components. This allows for a holistic evaluation, rather than a comparison of different components, and ensures that all participants would receive the benefits of the entire intervention. While extensive research has been conducted on barriers to mental health help seeking amongst university students, there is a lack of such studies done within the Singaporean context. To the best of our knowledge, this is the first study in Singapore that examines the effects of an anti-stigma intervention on bystander intervention. This study, being the first of its kind in Singapore, paves the way for the future examination of longer-term effects of such interventions on university students. It also allows for the potential extrapolation of these effects onto other population subgroups, and even the Singaporean population.

There are several limitations to our study. First, the low number of items in the BISD violates the assumption of tau-equivalence, which may explain the low Cronbach’s alpha values (52). Second, using convenience sampling resulted in a sample that was not ethnically representative of the Singapore population, thereby limiting the comparisons that can be made between ethnic groups. Participation was also voluntary, so students who took part may already have better attitudes or knowledge on the subject matter. Third, the lack of a control group meant that it would be difficult to differentiate whether the change in bystander attitudes resulted solely from the ARTEMIS intervention or other unaccounted factors, and any results from the study should thus be interpreted with this in mind. While we do not expect other factors to have a significant effect on participants’ bystander attitudes, the inclusion of a control group would have certainly strengthened this notion. Fourthly, the first follow-up was done immediately post-intervention whilst the second one was done after 3 months, which could result in participants dropping out for various reasons such as a lack of availability or a loss of interest. This introduces the possibility of attrition bias, which may have caused the sample to characteristically differ at different time points. Lastly, this study was only conducted in one local university. Given that different universities may have different institutional or campus cultures, the applicability of these results may be limited.

## Conclusion

In conclusion, ARTEMIS led to promising short-term results across BISD factors. In the short run, those who participated reported being more likely to intervene when their peers suffer from depression. However, these results were not sustained in the long term, suggesting the need for ‘booster’ interventions to reinforce the short-term benefits. Given ARTEMIS’s single-intervention session design, more research is needed to evaluate any possible long-term effects when more sessions are included. Several directions for future research include (1) replicating this study in other local universities to examine effects across institutional or campus cultures; (2) examining bystander intervention for different mental illnesses; and (3) exploring the long-term impacts of multiple-session interventions or ‘booster’ interventions on attitudes toward mental illness.

## Data Availability

The original contributions presented in the study are included in the article/**Supplementary Material**. Further inquiries can be directed to the corresponding author.
