# Supplementary material for: Examining bystander intervention for peer depression and sociodemographic correlates among university students in Singapore
Source: Front Psychiatry. 2024 Aug 28;15:1307807. doi: 10.3389/fpsyt.2024.1307807 (PMC11388726; doi:10.3389/fpsyt.2024.1307807)
Supplement: Supplementary file 1 [file Table1.docx]

Examining bystander intervention for peer depression and sociodemographic correlates among university students in Singapore

**Appendix**

**Cronbach’s alpha values for the BISD factors (14 included items only)**

|  | Pre-intervention | Post-intervention | 3 month-intervention |
| --- | --- | --- | --- |
| Factor 1: Awareness of depression among peers | 0.611 | 0.606 | 0.561 |
| Factor 2: Vigilance towards possible symptoms of depression | 0.491 | 0.618 | 0.618 |
| Factor 3: Knowledge on how to intervene | 0.746 | 0.681 | 0.793 |
| Factor 4: Acceptance of responsibility to intervene | 0.742 | 0.834 | 0.796 |

| **BISD \|** The table of BISD items, taken from Ong et al. (2021) |
| --- |
| *Factor 1: Awareness of depression among peers* |
| I am aware that there are students at my university who are experiencing depressive symptoms |
| I have seen students showing signs of depression at my university |
| It is evident to me that someone who is experiencing depressive symptoms needs support |
|  |
| *Factor 2: Vigilance towards possible symptoms of depression* |
| If a peer withdraws from activities that they usually enjoy, they may just be tired (Reverse scoring) |
| If someone tells me that they feel hopeless about the future, I will think that it is a phase that everyone goes through (Reverse scoring) |
|  |
| *Factor 3: Knowledge on how to intervene* |
| I know what to say to support a student who is experiencing depression |
| I know who to refer a peer who show signs of depression for help |
| I know who to alert when a peer is in crisis |
| I would inform the university’s faculty if a peer is showing worsening signs of depression and is reluctant to seek help |
|  |
| *Factor 4: Acceptance of responsibility to intervene* |
| If I am aware that a peer is showing signs of depression, I feel it is my responsibility to help the person |
| I believe that my actions can have a positive impact on a peer who is having depression |
| If I saw a peer who I did not know very well showing signs of depression, I would help them |
| If I notice a peer with signs of depression, I will ask them how things are going |
| If I know a peer who shows signs of depression, I would offer to accompany him to seek help |
|  |
| *Items removed* |
| I know what are the signs and symptoms of depression |
| I don’t think there is anything I can do to help a peer who is having depression |
| I feel that other students are in a better position to help a peer who is showing signs of depression |
